# Content validation of the nursing diagnosis “inadequate social support network”

**DOI:** 10.1590/1980-220X-REEUSP-2023-0250en

**Published:** 2024-02-09

**Authors:** Michelline Santos de França, Cleide Maria Pontes, Marcos Venícios de Oliveira Lopes, Ryanne Carolynne Marques Gomes Mendes, Jaqueline Galdino Albuquerque Perrelli, Sheila Coelho Ramalho Vasconcelos Morais, Francisca Márcia Pereira Linhares

**Affiliations:** 1 Instituto Federal de Pernambuco, Departamento de Enfermagem, Abreu e Lima, PE, Brazil.; 2 Universidade Federal de Pernambuco, Departamento de Enfermagem, Recife, PE, Brazil.; 3 Universidade Federal do Ceará, Departamento de Enfermagem, Fortaleza, CE, Brazil.

**Keywords:** Social Support, Nursing Diagnosis, Validation Study, Social Network, Nursing Process, Apoyo Social, Diagnóstico de Enfermería, Estudio de Validación, Red Social, Proceso de Enfermería, Apoio Social, Diagnóstico de Enfermagem, Estudo de Validação, Rede Social, Processo de Enfermagem

## Abstract

**Objective:**

To evaluate evidence of content validity of the nursing diagnosis “inadequate social support network”.

**Method:**

A methodological study of the content validation type, carried out with 23 judges who evaluated the adequacy of the title, definition, class and domain of the nursing diagnosis “inadequate social support network”. The judges also assessed the relevance of 28 clinical indicators and 32 etiological factors, which were considered valid when the Content Validity Index was ≥ 0.9.

**Results:**

The judges agreed with the proposed title and suggested changes to the definition of the nursing diagnosis. They recommended its inclusion in Domain 7 – “Roles and relationships” and Class 3 – “Role performance” of the NANDA-I taxonomy. In addition, 19 clinical indicators and 27 etiological factors were considered relevant.

**Conclusion:**

The nursing diagnosis “inadequate social support network” had its theoretical structure validated in terms of content, which can support the practice of nurses in the operationalization of the Nursing Process.

## RESUMO

**Objetivo:**

Avaliar evidências de validade de conteúdo do diagnóstico de enfermagem “rede social de apoio inadequada”.

**Método:**

Estudo metodológico do tipo validação de conteúdo, realizado com 23 juízes que avaliaram a adequação do título, da definição, da classe e do domínio do diagnóstico de enfermagem “rede social de apoio inadequada”. Os juízes também avaliaram a relevância de 28 indicadores clínicos e de 32 fatores etiológicos, os quais foram considerados válidos quando o Índice de Validade de Conteúdo foi ≥ 0,9.

**Resultados:**

Os juízes concordaram com o título proposto e sugeriram alterações na definição do diagnóstico de enfermagem. Recomendaram a sua inserção no Domínio 7 – “Papéis e relacionamentos” e na Classe 3 – “Desempenho de papéis” da taxonomia da NANDA-I. Ademais, 19 indicadores clínicos e 27 fatores etiológicos foram considerados relevantes.

**Conclusão:**

O diagnóstico de enfermagem “rede social de apoio inadequada” teve sua estrutura teórica validada quanto ao conteúdo, a qual pode subsidiar a prática do enfermeiro na operacionalização do Processo de Enfermagem.

## RESUMEN

**Objetivo:**

Evaluar las evidencias de la validez de contenido del diagnóstico de enfermería “red de apoyo social inadecuada”.

**Método:**

Estudio metodológico de tipo validación de contenido, realizado con 23 jueces que evaluaron la adecuación del título, definición, clase y dominio del diagnóstico de enfermería “red de apoyo social inadecuada”. Los jueces también evaluaron la pertinencia de 28 indicadores clínicos y 32 factores etiológicos, que se consideraron válidos cuando el Índice de Validez de Contenido fue ≥ 0.9.

**Resultados:**

Los jueces estuvieron de acuerdo con el título propuesto y sugirieron cambios en la definición del diagnóstico de enfermería. Recomendaron su inclusión en el Dominio 7 - “Roles y relaciones” y en la Clase 3 - “Desempeño de roles” de la taxonomía NANDA-I. Además, se consideraron relevantes 19 indicadores clínicos y 27 factores etiológicos.

**Conclusión:**

El diagnóstico de enfermería “red de apoyo social inadecuada” tuvo su estructura teórica validada en términos de contenido, lo que puede apoyar la práctica del personal de enfermería en la operacionalización del Proceso de Enfermería.

## INTRODUCTION

The interactions between individuals and the members of their social support network have a positive or negative impact on their lives. The strengthening of established ties provides the necessary support in the various events of the life cycle. On the other hand, fragile relationships can lead to a diminished sense of belonging, a shortage of sources of support and, consequently, an inadequate social support network^(1)^.

An inadequate social support network can have repercussions on the health-disease process, and it is up to nurses to be aware of the interactive possibilities of the individuals they care for. This professional can recognize themselves as a member of the network, establishing links and relationships in an attitude of co-responsibility for comprehensive care. In addition, they can develop health education actions that value people’s life stories and prior knowledge, in a process of joint dialogical construction^(2)^.

Such personal involvement actions are more common in Primary Health Care, which provides longitudinal care, in which nurses play a fundamental role, and whose therapeutic relationship is built on responsibility and trust in the continuous monitoring of health problems and preventive actions by the same health team over time^(3)^.

Against this backdrop, establishing the nurse’s bond with the family and the social support network is common when caring for patients from specific groups, such as people with mental disorders, disabilities, obesity and chronic diseases. Although it is more feasible in the cases mentioned above, the establishment of a nurse-patient bond can and should also occur in other settings, such as hospital sectors (maternity wards, oncology wards, intensive care units and surgical wards), where an inadequate social support network can be diagnosed and interventions should be prescribed and carried out. Thus, the person’s interactions with their social support network, as well as the presence and adequacy of the support offered to them, should be the subject of investigation and intervention by nurses^(2)^.

In this sense, an inadequate social support network can be understood as a nursing phenomenon, and it is necessary to define parameters for its accurate identification. Understanding this nursing phenomenon requires understanding the mechanisms of how it is established and manifests itself in people’s lives, and enables the implementation of interventions that can minimize its occurrence^(4)^.

Translating a phenomenon into a Nursing Diagnosis (ND) contributes to the consolidation of nursing science and the delimitation of its unique body of knowledge for assisting patients in their responses to health problems and/or life processes^(4)^. From this perspective, the proposal for a new ND was structured, initially called “ineffective social support network”, whose judgment was later changed to “inadequate”. This new ND highlights the complexity of interpersonal relationships and their impact on life processes. By being incorporated into the scope of nursing through standardized language, it will make it possible to expand praxis and reflect on theoretical models of comprehensive care for the person, family and human community.

In the process of structuring the new ND, theoretical procedures were carried out to elucidate the phenomenon through the development of a predictive Mid-Range Theory, and the validation of the ND to identify and evaluate the etiological factors (antecedents) and the defining characteristics (consequents) indicative of the phenomenon.

Among the ND validation methods, content validation stands out, which ensures that the knowledge of the nursing phenomenon is defined and exposed to evaluation by judges with expertise in the subject. This evaluation enables the composition of the theoretical diagnostic structure, which must then be submitted to clinical validation in a population potentially exposed to the phenomenon^(5)^.

This research is essential, since the elements of the ND “inadequate social support network” need to be refined and improved, and will contribute to diagnostic reasoning in care, research and teaching and, consequently, to the planning of nursing care and the implementation of interventions to promote people’s social support network. The results will contribute to advances in the field of nursing and support the identification of ND, which is a phenomenon that can be found at the primary, secondary and tertiary levels of health care.

The aim of this study was to evaluate evidence of the content validity of the nursing diagnosis “inadequate social support network”.

## METHOD

### Type of Study

Methodological study of the content validation type, of the ND “inadequate social support network” according to the NANDA-I taxonomy. Diagnosis validation studies aim to verify with judges the relevance of clinical indicators and etiological factors as components of an ND; and to ascertain the clarity and precision of the conceptual and operational definitions of these elements, and of the definition of the ND^(5)^.

### Location

Conducted via e-mail with nurse judges in Brazil.

### Population and Selection Criteria

The population consisted of nurse judges. The criteria for selecting the judges were: 1) having clinical and/or research experience in the area of social support networks and/or ND, defined by at least two years of experience in direct care for individuals, families and communities, with identification of ND; and/or publishing at least two academic papers (articles, abstracts in conference proceedings, book chapters or books) on the subject of social support networks and/or ND; and 2) being a nurse with a minimum master’s degree or studying for a master’s or doctorate in the area of ND and/or social support networks. Judges who met at least one of the selection criteria were included. Those who did not respond to the invitation letter were excluded.

### Sample Definition

The sample was defined based on the estimated average of the evaluations - Content Validity Index (CVI) – for each element to be analyzed. The confidence level (*Z*_1_–α/2) was 95%, the standard deviation (S) was 0.17 and the sampling error (e) was 0.07. Thus, using the formula: n0 = (*Z*_1_–α/2.s/e)2, the sample totaled 23 nurse judges.

### Data Collection

Predictive diversity was used for data collection, which assumes that the accuracy of a given group’s inferences is directly proportional to the diversity of its members’ level of expertise. The collective wisdom approach was also used, which defines that the opinion of a group of judges has better estimates than that of a single judge. This technique is based on the idea that each individual, regardless of their level of expertise, can make mistakes. However, when you have the averages of the answers, the error is canceled out (the average obtained in the group is greater than the judgment of just one individual)^(5)^.

The definition of the level of expertise comes from the academic knowledge and clinical experience of the judges^(5,6)^. For this definition, the following classification was used: novice, advanced beginner, competent, proficient and expert^(6,7)^.

Data was collected from the judges between July and October 2018. The judges were recruited through the Lattes platform of the National Council for Scientific and Technological Development (CNPq). An advanced CV search was used in the databases of doctors and other researchers, with the help of the terms “social network” and “nursing diagnosis”. The judges obtained via the platform also indicated other professionals, who were able to make up the sample after applying the inclusion and exclusion criteria.

Each judge selected received an invitation letter via email. Those who agreed to take part in the study received the Informed Consent Form (ICF) and the data collection instrument. This had to be answered and returned within 15 days.

The data collection instrument contained a presentation of the study and instructions on how to fill it in properly, and consisted of three parts. The first referred to the characterization of the judges; the second contained questions about the appropriateness of the title, definition, domain and class, according to the NANDA-I taxonomy^(4)^. In the third part, there were 28 clinical indicators and 32 etiological factors of the ND under study, with their respective conceptual and operational definitions, which were drawn up using scientific articles from literature reviews, textbooks, dictionaries, theses and dissertations.

Each clinical indicator and etiological factor was assessed for relevance using a five-point Likert scale (totally irrelevant, slightly relevant, partially relevant, very relevant and totally relevant). In addition, there was space in all of them to consider the clarity and precision of the definitions. When the judges felt that the definition already met the criteria for clarity and precision, they left the space blank.

The nursing diagnosis under study was initially drafted under the title “Ineffective social support network”, but NANDA-I, in agreement with the authors of the ND, opted for its inclusion in the taxonomy by changing the term “ineffective” to “inadequate” in order to adhere to the process of standardizing the terms of this taxonomy.

### Data Analysis and Processing

The data was tabulated in a Microsoft Office Excel 2010 spreadsheet and analyzed using the Statistical Package for the Social Sciences (SPSS), version 21; and R software, version 3.2.0. Descriptive analysis was carried out, including the calculation of frequencies and 95% Confidence Intervals (CI) for nominal variables. Quantitative variables were presented using the mean, median, standard deviation and interquartile range. The Shapiro-Wilk test was applied to check that the data adhered to the normal distribution.

Data on the relevance of the ND components was analyzed by calculating the CVI, based on the predictive diversity model, and the judges’ evaluation was weighted by their level of expertise. Since the CVI estimates did not adhere to the normal distribution using the Shapiro-Wilk test, the medians of the evaluations were used, with the CI of the CVI median ≥ 0.9 as the reference value. Clinical indicators and etiological factors with median CVI values for the relevance criterion below 0.9 were excluded. The criteria of clarity and objectivity of the conceptual and operational definitions were assessed qualitatively by the judges, who could make observations and suggestions for changes when they deemed it necessary. The suggestions were verified by the authors and the relevant changes were made to the definitions.

### Ethical Aspects

The study was conducted in accordance with the guidelines of Resolution no. 466/2012 and cleared by the Research Ethics Committee of the Federal University of Pernambuco, according to final opinion no. 4.471.097 in 2020. Participants’ consent was obtained by signing the informed consent form.

## RESULTS

Of a total of 23 judges, the majority (69.6%) had a doctorate and only one was male. The majority were from the Northeast (69.6%), followed by the South (21.7%) and Southeast (8.7%). The majority (91.3%) worked as teachers and the area of study with the highest degree was ND (52.2%) and the social support network (47.8%). In addition, 39.1% of the judges were classified as competent, 17.4% as proficient and 8.7% as expert.

The average age of the judges was 38.96 years and the average number of years working in research groups on nursing terminology was 6.67 years. With regard to the length of time the judges have been trained, the median is 11 years; in addition to three years for the length of time they have worked in social support networks; six years for the length of time they have worked identifying NDs; six years for the length of time they have taught the subject of NDs; and two and a half years for the length of time they have worked in research groups on social support networks.

Regarding the appropriateness of the proposed title (inadequate social support network) for ND, only one judge flagged it as inappropriate. However, he made no suggestions for changes, so the proposed title was maintained. Of the three diagnostic definition options presented, Definition 2 – “Inability of network members to establish social interactions that generate support to meet the needs of the person, family and community” was chosen by 56.5% of the judges, followed by Definition 1 – “Network of interpersonal contacts and organizational systems unable to mobilize to provide the necessary support”, chosen by 30.4% of the judges, as described in Table 1.

**Table 1:** Evaluation of the adequacy of the definition of the Nursing Diagnosis “inadequate social support network” – Recife, PE, Brazil, 2018.

Definition	n = 23	%
Definition 1 – Network of interpersonal contacts and organizational systems unable to mobilize to provide the necessary support.	7	30.4
Definition 2 – Inability of network members to establish social interactions that generate support for meeting the needs of the person, family and community.	13	56.5
Definition 3 – Inability of network members to establish social interactions that provide support.	3	13.0
Total	23	100

n = Sample size; % – Percentage.

There was no consensus among the judges on the most appropriate definition, but they did make suggestions, which were accepted and resulted in a new definition that encompasses the aspects present in the suggested options, namely: “Inability of the members of the interpersonal contact group and organizational systems to establish social interactions that generate support for meeting the needs of the person, family and community”. In addition, the inclusion of the ND “inadequate social support network” in Domain 7 – “Roles and relationships” and in Class 3 – “Role performance” was recommended by 95.7% of the judges.

Of the 28 clinical indicators, nine had a CVI value below 0.9, namely: Low situational self-esteem, Conditioned support, Scarcity of possible sources of social support, Excess of insignificant interpersonal contacts, Little social support, Perception of normality of the person’s problem, Excess of information and interaction from the social network, Lack of material goods and Depression, as described in Table 2.

**Table 2:** Validity of the clinical indicators of the Nursing Diagnosis “inadequate social support network” – Recife, PE, Brazil, 2018.

Clinical Indicators	Shapiro-Wilk test		CVI		
	W	p-value	Median	CI 95%	
Feeling abandoned	0.33	<0.001	1.00	1.00	1.00
Decreased emotional support	0.45	<0.001	1.00	1.00	1.00
Inadequate information support	0.27	<0.001	1.00	1.00	1.00
Lack of instrumental support from health services	0.24	<0.001	1.00	1.00	1.00
Imposition of appropriate behavior	0.62	<0.001	1.00	0.75	1.00
Support offered different from that expected	0.46	<0.001	1.00	1.00	1.00
Fragile bonds	0.39	<0.001	1.00	1.00	1.00
Negative social interactions	0.45	<0.001	1.00	1.00	1.00
Prejudice	0.44	<0.001	1.00	1.00	1.00
Neglect of demands for support	0.45	<0.001	1.00	1.00	1.00
Encouragement of negative behavior	0.45	<0.001	1.00	1.00	1.00
Underestimation of the person’s self-efficacy	0.56	<0.001	1.00	0.88	1.00
Blaming attitudes	0.42	<0.001	1.00	1.00	1.00
Invasion of privacy	0.46	<0.001	1.00	1.00	1.00
Loss of confidentiality	0.33	<0.001	1.00	1.00	1.00
Overburdening the main caregiver	0.34	<0.001	1.00	1.00	1.00
Unwillingness to offer social support	0.53	<0.001	1.00	0.87	1.00
Devaluation of the social support received	0.47	<0.001	1.00	1.00	1.00
Low reciprocity	0.58	<0.001	1.00	0.63	1.00
Low situational self-esteem	0.6	<0.001	0.88	0.88	1.00
Conditioned support	0.65	<0.001	0.88	0.87	1.00
Scarcity of possible sources of social support	0.6	<0.001	0.88	0.87	1.00
Too many insignificant interpersonal contacts	0.63	<0.001	0.88	0.75	1.00
Little social support	0.66	<0.001	0.87	0.75	1.00
Perception that the person’s problem is normal	0.68	<0.001	0.75	0.75	1.00
Excess of information and interaction of the social network	0.65	<0.001	0.75	0.75	1.00
Lack of material goods	0.68	<0.001	0.63	0.5	0.88
Depression	0.74	<0.001	0.5	0.5	0.5
ALL CI	0.79	<0.001	0.75	0.75	0.87

CVI – Content Validity Index; ALL CI – All the Clinical Indicators.

Of the 32 etiological factors submitted for analysis, 27 were considered relevant by the judges. The five etiological factors judged not to be relevant to the ND were: Larger social network, Mental health problems, Consumption of the person’s resources, High degree of complexity of organizations and Unexpected events, as described in Table 3.

**Table 3:** Validity of the etiological factors of the Nursing Diagnosis “Inadequate social support network” – Recife, PE, Brazil, 2018.

Etiological factors	Shapiro-Wilk test		CVI		
	W	p-value	Median	CI 95%	
Low social network density	0.3	<0.001	1.00	1.00	1.00
Smaller social network	0.49	<0.001	1.00	1.00	1.00
Insufficient commitment from health professionals	0.33	<0.001	1.00	1.00	1.00
Impersonal relations between health professional and patient	0.51	<0.001	1.00	0.88	1.00
Intrusive practices by health professionals	0.53	<0.001	1.00	0.88	1.00
Weak organization of institutional services into a network	0.38	<0.001	1.00	1.00	1.00
Shortage of health professionals	0.49	<0.001	1.00	0.88	1.00
Difficulties in geographical access to secondary social network services	0.41	<0.001	1.00	1.00	1.00
Excessive demand for support	0.26	<0.001	1.00	1.00	1.00
Fear of the person seeking support	0.42	<0.001	1.00	1.00	1.00
Mistrust of the other person’s competence	0.5	<0.001	1.00	1.00	1.00
Lack of sociability	0.22	<0.001	1.00	1.00	1.00
Refusal of support	0.22	<0.001	1.00	1.00	1.00
Unable to access the secondary social network services	0.45	<0.001	1.00	1.00	1.00
Social isolation	0.42	<0.001	1.00	1.00	1.00
Moving house	0.38	<0.001	1.00	1.00	1.00
Cultural differences	0.22	<0.001	1.00	1.00	1.00
Centralization of responsibility for care	0.38	<0.001	1.00	1.00	1.00
Restriction of the role of a member of the social network	0.33	<0.001	1.00	1.00	1.00
Disregard for the role of supporter	0.55	<0.001	1.00	0.88	1.00
Lack of strong ties	0.42	<0.001	1.00	1.00	1.00
Broken ties	0.26	<0.001	1.00	1.00	1.00
Dependency-generating ties	0.34	<0.001	1.00	1.00	1.00
Lack of recognition of the support offered	0.41	<0.001	1.00	1.00	1.00
Lack of reciprocity for the support received	0.34	<0.001	1.00	1.00	1.00
Unwillingness to support	0.55	<0.001	1.00	0.88	1.00
Lack of theoretical and practical knowledge about the person’s needs	0.49	<0.001	1.00	0.88	1.00
Larger social network	0.65	<0.001	0.88	0.75	1.00
Mental health problems	0.61	<0.001	0.88	0.63	1.00
Consumption of the person’s resources	0.6	<0.001	0.88	0.75	1.00
Unexpected events	0.63	<0.001	0.88	0.75	1.00
High degree of complexity in organizations	0.73	<0.001	0.75	0.50	0.87
ALL EF	0.73	<0.001	0.87	0.87	0.88

CVI – Content Validity Index; ALL EF – All the Etiological factors.

The judges proposed changes to the titles of some of the clinical indicators, which allowed for better textual fluidity, intelligibility, removal of repetitive texts and changes to the titles of some indicators. The following title changes were suggested and accepted: “Little social support” for “Decreased social support”; “Encouragement of negative behavior” for “Encouragement of negative behavior”; and “Underestimation of the person’s self-efficacy” for “Underestimation of self-efficacy”.

The conceptual and/or operational definitions were adjusted in the indicators: Decreased social support, Devaluation of the support received, Feeling of abandonment, Decreased emotional support, Deficit of instrumental support from health services, Underestimation of the person’s self-efficacy and Overload of the main caregiver.

In relation to the etiological factors, suggestions were made to change the title, which were accepted, namely: “Weakness of the organization of institutional services in a network” to “Weakness of the organization of institutional services in a network”. The conceptual and/or operational definitions of the following factors were also altered: Smaller social network, Insufficient commitment on the part of health professionals, Cultural differences, Lack of recognition of the support offered and Lack of theoretical and practical knowledge about the person’s needs.

The changes made to the conceptual and operational definitions of the clinical indicators and etiological factors as a result of the judges’ qualitative assessment were textual in nature and contributed to improving the clarity and precision of the elements. These changes were particularly concentrated on the operational definitions, so that they expressed a single concept, and the exchange of verbs for others that represented actions that could be verified at the time of clinical validation of the ND.

## DISCUSSION

This study used the collective wisdom approach, which assumes that, regardless of their level of expertise, judges can make clinical judgment errors, which are minimized by predictive diversity^(5)^. Thus, the opinion of the judges, combined with the authors’ capacity for discernment in accepting suggestions, makes it possible to present a cohesive theoretical structure resulting from the collaboration of the judges, who did not participate in the initial construction, but who infused their perceptions during the validation process described.

It should be noted that the whole process of developing new NDs contributes to strengthening nursing science, as it broadens the spectrum of cataloging phenomena of interest^(4)^ such as the inadequate social support network. In this sense, the recognition of the potential for interpersonal relationships to interfere in the health-disease process, as well as the development of standardized terminology, configures nursing’s connection with this theme.

The title proposed for the ND “inadequate social support network” is based on the nursing phenomenon of the ineffectiveness of the social support network, which often contributes to difficulties in achieving good health outcomes^(1,2)^. Individuals, families and collectivities are inserted into networks with problems in their structure, with few members, relationships established on fragile or broken bonds, members who do not play the role of supporters, immersion in an environment of tensions and an excess of negative interactions^(2)^.

In view of this, the new definition constructed on the basis of the judges’ suggestions covers the phenomenon of nursing ineffectiveness of the social support network in its entirety, from the nuances of the primary and secondary networks to incompetence in fulfilling the support function. Providing social support is the network’s main mission^(1)^. Therefore, the definition of the phenomenon that represents its ineffectiveness must take into account the difficulties in performing this function.

Clinical indicators such as “Feeling abandoned” and “Devaluing the support received” can be seen by the nurse in the person being cared for, as in the case of women experiencing breastfeeding for the first time. Nursing mothers may feel abandoned when faced with unavailable services or health care linked exclusively to the biomedical model, permeated by excessive judgment, to the detriment of social relationships, which leads to a lack of a sense of belonging and the feeling of not having anyone to count on, since the person stops going to health services where they don’t feel welcomed, and this contributes to maintaining the feeling of abandonment. The devaluation of the support received, in turn, manifests itself in situations where the support offered by the social support network is authoritarian and discrepant from what was expected^(8)^.

For these indicators, changes were suggested in the conceptual and/or operational definitions, which improved the intelligibility of the indicators, with the use of frequent and standardized vocabulary in the NANDA-I taxonomy, as well as the elimination of redundancies, essential for communicative precision and risk-free nursing care^(9)^.

Improving the description of instrumental support in the clinical indicator “Deficit in instrumental support from health services” ensured the clarity of this component. Direct help of a practical nature can be translated into actions and materials to solve the problems presented by the network that make it easier to carry out tasks^(10)^. This type of support, coming from the health services in which nursing operates, is particularly relevant for people in vulnerable situations, as observed in a study of elderly Japanese people in which those who received instrumental support were less likely to have unmet health needs^(10)^; or people who are geographically distant from their supporters, according to a study of Irish immigrants living in London, which showed that immigrants who could count on at least three people and received support in times of crisis were more likely to have a good self-rated health^(12)^.

The etiological factor “Smaller social network” shows the existence of a few members who are responsible for providing all the essential support. These networks are usually characterized by few family members, friends, neighbors and low social participation. The limited number of members in the network usually deteriorates the availability of support^(13,14)^ and, like many social characteristics, should be investigated by nurses based on the person’s perception, a recommendation included in the operational definition.

The change in the operational definition of the etiological factor “Insufficient commitment on the part of health professionals”, with the inclusion of the expression “report of”, made this element subject to evaluation, with a view to identifying its presence or absence. Health professionals, especially nurses, must recognize themselves as part of the person’s social support network, and are co-responsible for providing care and mobilizing other members of the network to meet demands^(8)^. However, the possible lack of involvement makes the already disjointed network even more inadequate and reinforces stigmas^(15)^.

The addition of the terms “habits and beliefs” to the definition of the etiological factor “Cultural differences” helped to explain more precisely what is meant by culture. The lack of cultural sensitivity and respect for differences that permeates interaction and communication without the purpose of support presupposes the ineffectiveness of the social support network, which is reflected in people and social groups who receive insufficient support and are exposed to greater health risks^(13)^.

“Lack of recognition of the support offered” represents situations in which support is offered by members of the network, but is not identified by the person. The perception of the support offered in different contexts results in satisfaction with life and gratitude towards those who offer it^(16)^. This drives reciprocity, the lack of which means little mutuality in relationships, which become a one-way street and contribute to the fatigue of supporters.

These relationships should be investigated by nurses in clinical practice, as well as the “deficit of theoretical and practical knowledge about the person’s needs”. Competencies are sets of knowledge and skills which, in turn, are the qualities needed to perform a certain activity^(17)^. Members of the social support network need to have some kind of skill in order to offer timely help with the person’s specific needs; therefore, “skill” is the more appropriate term, rather than “competence”.

The description of the process of validating the content of the ND “inadequate social support network” helps nurses to understand the procedure for designing each of its elements. In this way, it is possible to better understand and clarify how the inadequate social support network manifests itself, and to translate this knowledge into nurses’ diagnostic reasoning in the different contexts of care for the person, family and community.

A limitation of this study is the composition of the sample, with a predominance of females and teachers. Furthermore, there was little regional variability among the participating judges.

As an advance for the nursing field, the ND “inadequate social support network” reflects the professional domain of nursing, by presenting the antecedents and consequences of the phenomenon, which affects people in a variety of situations, especially in times of need. Broadening the focus of attention to the community represented by the social support network contributes to improving nursing care, which now involves interpersonal contacts in nursing care and health education.

## CONCLUSION

The ND “inadequate social support network” has adequate evidence of content validity and was accepted for inclusion in the Classification of Diagnoses of the NANDA-I taxonomy (2024–2026). The judges’ suggestions were fundamental in reformulating the definition of the diagnosis, as well as making adjustments to the clarity and precision of the conceptual and operational definitions of the 19 clinical indicators and 27 etiological factors considered relevant.

This new ND will enable the development of nursing interventions with the integration of all members of the social support network, in order to obtain results based on the autonomy and co-responsibility of the members of the network. It is recommended that clinical research be carried out in order to identify the ND “inadequate social support network” in susceptible populations and in different settings.
